# Mixotrophic Cultivation Optimization of Microalga *Euglena pisciformis* AEW501 for Paramylon Production

**DOI:** 10.3390/md20080518

**Published:** 2022-08-14

**Authors:** Panpan Fan, Yanhua Li, Rui Deng, Feixia Zhu, Fengfeng Cheng, Gaofei Song, Wujuan Mi, Yonghong Bi

**Affiliations:** 1 State Key Laboratory of Freshwater Ecology and Biotechnology, Institute of Hydrobiology, Chinese Academy of Sciences, Wuhan 430072, China; 2 University of Chinese Academy of Sciences, Beijing 100049, China; 3 College of Life Sciences, Xinjiang Normal University, Urumqi 830054, China; 4 College of Fisheries and Life Science, Dalian Ocean University, Dalian 116023, China

**Keywords:** *Euglena pisciformis* AEW501, mixotrophic cultivation, nutritional components analysis, paramylon, characterization

## Abstract

*Euglena*, a flagellated unicellular protist, has recently received widespread attention for various high-value metabolites, especially paramylon, which was only found in Euglenophyta. The limited species and low biomass of *Euglena* has impeded paramylon exploitation and utilization. This study established an optimal cultivation method of *Euglena pisciformis* AEW501 for paramylon production under mixotrophic cultivation. The results showed that the optimum mixotrophic conditions were 20 °C, pH 7.0, and 63 μmol photons m^−2^∙s^−1^, and the concentrations of sodium acetate and diammonium hydrogen phosphate were 0.98 g L^−1^ and 0.79 g L^−1^, respectively. The maximal biomass and paramylon content were 0.72 g L^−1^ and 71.39% of dry weight. The algal powder contained more than 16 amino acids, 6 vitamins, and 10 unsaturated fatty acids under the optimal cultivation. *E. pisciformis* paramylon was pure β-1,3-glucan-type polysaccharide (the purity was up to 99.13 ± 0.61%) composed of linear glucose chains linked together by β-1,3-glycosidic bonds. These findings present a valuable basis for the industrial exploitation of paramylon with *E. pisciformis* AEW501.

## 1. Introduction

Over the past decades, microalgal biomass as a natural resource for producing high-value metabolites has been available on the global market in a variety of commercial products [1]. The global market of microalgal merchandise has been estimated to attain USD 1143 million by 2024 [2]. The common high-value microalga on the market include *Chlorella vulgaris* [3], *Spirulina maxima* [4], *Haematococcus pluvialis* [5], *Dunaliella salina* [6], and *Euglena gracilis* [7]. *Euglena*-based productions with unique animal cell properties have better absorption as a food supplement [8]. Additionally, the unique paramylon in the *Euglena* cell has various active functions, such as anti-inflammatory [9], antitumor [10], immunomodulation [11], liver protection [12], and wound healing [13].

The potential of *Euglena* used as a paramylon source has gained increased attention in recent years. The free paramylon granule is a β-1,3-glucan, which is deposited as a storage polysaccharide in the cytoplasm of Euglenoids [14]. *Euglena* paramylon is a valuable commercial product due to the biologically active function (such as immune support) determined by the linear β-1,3-glucan structure [15]. Physical and chemical methods and algae–bacteria co-culturing have been used to increase paramylon production. Previous research reported that platinum electrode electrical treatment under 10 mA current intensity on *E. gracilis* cells had a significant effect on paramylon accumulation [16]. Additionally, salt stress could induce paramylon accumulation in *E. gracilis* cells [17]. *E. gracilis* could achieve higher paramylon production in a co-culture system of *E. gracilis* and bacterium [18,19,20]. Notably, paramylon content was enhanced when *E. gracilis* cells were transferred from mixotrophic to heterotrophic conditions [21]. All these studies focused on *E. gracilis*, and it is necessary to exploit other species of *Euglena* with a high yield of paramylon to meet the huge market demands due to the limitation of algal biomass.

*E. pisciformis* AEW501 is a strain of unicellular flagellate photosynthetic microalga isolated from fresh water in China. Studies indicated *E. pisciformis* has both plant characteristics of photosynthesis with chloroplasts and animal characteristics of using organic matter as a carbon source and swimming with flagella. According to the research by Flynn et al., *E. pisciformis* was termed “mixoplankton” [22]. *E. pisciformis* was mainly used for acute toxicity studies, and limited research on the application of cultivation and bioresource potential for the microalgal industry has been conducted [23,24]. However, the applications of *E. pisciformis* for biomass and high-value metabolite production have been progressively developed in recent years. It was shown that *E. pisciformis* AEW501 could grow under autotrophic, mixotrophic, and heterotrophic modes. Interestingly, the growth rate of this algae under mixotrophic conditions was higher and the paramylon granules were visible in the cytoplasm [25]. Therefore, mixotrophic cultivation is trending toward the development of new *Euglena* species to increase both biomass and paramylon production. Nevertheless, the cultivation condition was the bottleneck for the biomass, and the optimal mixotrophic cultivation method should be developed to meet the industrial demands.

Traditional β-1,3-glucans derived from plants that are mostly used in the food and pharmaceutical industries could not meet the needs of the market. However, β-1,3-glucans derived from microalga have been demonstrated to play an important role as free-radical scavengers and antioxidants for the prevention of oxidative damage in living organisms [11,26]. Therefore, microalga is considered to be the most promising bioresource of β-1,3-glucans especially *Euglena*. Ammonium chloride (NH_4_Cl) has been widely recognized as a fast-acting effective nitrogen source in microalga fields, and a previous study has reported that the utilization of NH_4_Cl both maximized the biomass production of *Poterioochromonas malhamensis* and also accumulated the storage of β-1,3-glucan [27]. However, urea was the most suitable nitrogen source for the growth of *Phaeodactylum tricornutum* [28], and the inorganic nitrogen source suitable for the growth of *Scenedesmus* was potassium nitrate [29]. However, the effect of different nitrogen sources on the biomass and paramylon production of *E. pisciformis* AEW501 is yet to be investigated.

In this study, we conducted the mixotrophic optimization cultivation of *E. pisciformis* AEW501 for paramylon production, analyzed the characterization analysis of paramylon, and revealed the structural characteristics of *E. pisciformis* paramylon. These findings provided a scientific basis for developing an effective large-scale commercial cultivation method of *E. pisciformis* for food supplements and pharmaceuticals.

## 2. Results

### 2.1. Optimal Mixotrophic Cultivation Method

**Figure 1:**
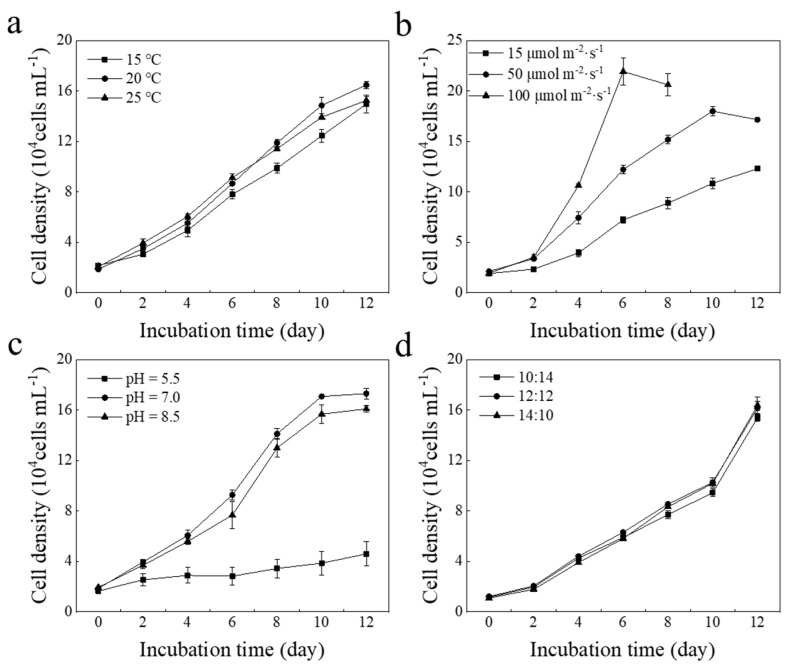
Environmental factor optimization for *E. pisciformis* AEW501. (**a**) Temperature; (**b**) light intensity; (**c**) pH; (**d**) light cycle. Data are shown as mean ± standard deviation (*n* = 3).

Cell density varied under different environmental conditions. As shown in Figure 1a, cell density accelerated with increasing temperature in the first 10 days; although there were no significant differences between the three treatments (*p* > 0.05), cell density was higher in the 20 °C treatment than in the 15 °C and 25 °C treatment groups at the end of cultivation. The light test indicated that cell density was notably higher in the 100 μmol photons m^−2^∙s^−1^ treatment than in other treatment groups (*p* < 0.05) but rapidly entered the decline phase due to the rapid lysis of the algal cell membrane (Figure 1b). Algal cells presented an exponential increase in the 50 μmol photons m^−2^∙s^−1^ treatment. The growth curves varied with different pH (Figure 1c). Algal biomass was significantly higher under the neutral and alkaline treatment groups than under the acidic treatment group (*p* < 0.05). The light cycle had no significant effect on cell growth (*p* > 0.05). In summary, the appropriate environmental conditions for the growth of *E. pisciformis* AEW501 have been 20 °C, 50 μmol photons m^−2^∙s^−1^, and an initial pH of 7.0.

**Figure 2:**
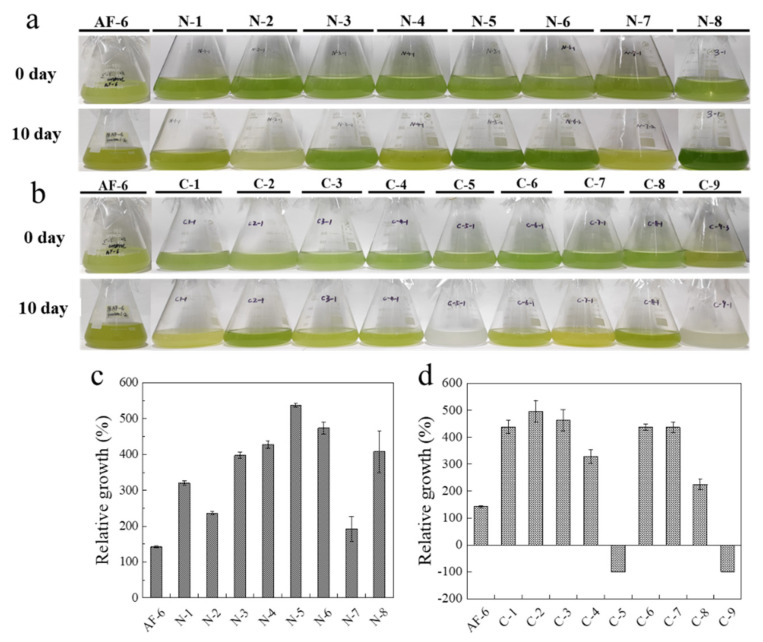
Nutritional factor optimization for *E. pisciformis* AEW501. (**a**) N-1 to N-8 represented sodium nitrate, urea, ammonium sulfate, tryptone, yeast extract, ammonium acetate, ammonium chloride, and diammonium phosphate. (**b**) C-1 to C-9 represented fructose, sodium acetate, ethanol, galactose, acetate, glycerin, glucose, sucrose, and α-Ketoglutaric acid. (**c**) Relative growth on different nitrogen sources after 14 days cultivation. (**d**) Relative growth on different carbon sources after 14 days cultivation. Data are shown as mean ± standard deviation of three replicates. Negative 100% of the relative growth represents complete death of algal cells in the corresponding treatments.

In nutritional optimization experiments, this study found that the chlorophyll content was higher in the yeast extract and diammonium phosphates treatment groups (Figure 2a). The medium supplementation with sodium nitrate, ammonium sulfate, tryptone, yeast extract, ammonium acetate, and diammonium phosphate could promote the relative growth of *E. pisciformis* AEW501 compared to the control medium (Figure 2c). The yeast extract treatment resulted in the highest specific growth rate, which was 1.96 times higher than the control medium. Diammonium phosphate was the most promising inorganic nitrogen, and the optimal concentration was 0.6 g L^−1^ (Figure S1a). Simultaneously, sodium acetate was the suitable organic carbon source for mixotrophic growth as it presented the highest specific growth rate compared to other organic carbon supplementation (Figure 2d). A further test demonstrated that the addition of 1.0 g L^−1^ sodium acetate could produce the maximal biomass (Figure S2a).

**Figure 3:**
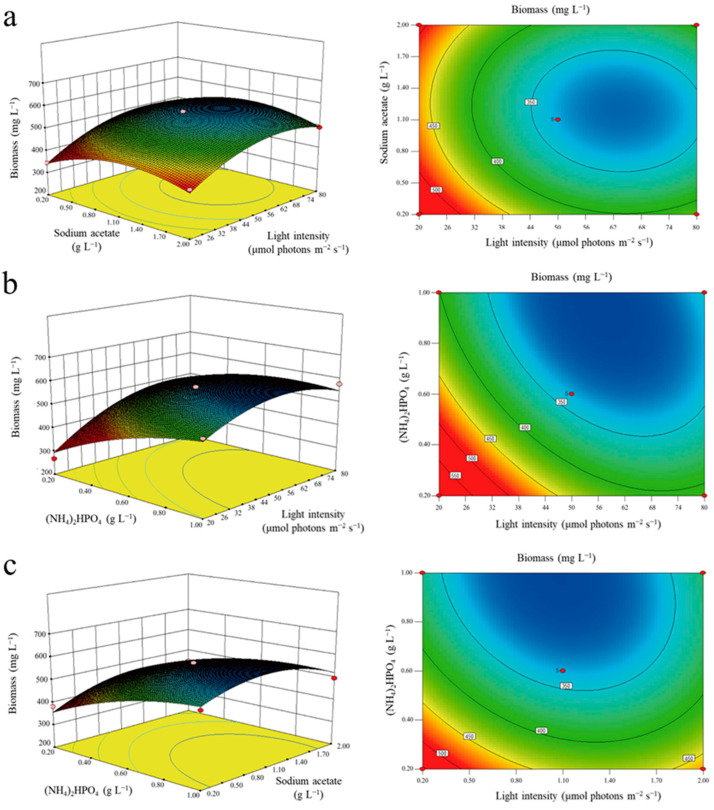
Surface and contour plots for the effect of light intensity and concentration of nutrition on the biomass of *E. pisciformis* AEW501. (**a**) Light intensity and concentration of sodium acetate; (**b**) light intensity and concentration of ammonium dihydrogen phosphate; (**c**) the concentration of sodium acetate and ammonium dihydrogen phosphate.

Three factors (light intensity, diammonium phosphate, and sodium acetate) and three levels were adopted for the Box–Behnken Design. The response surface of light intensity was steeper than that of the other two factors, indicating that light intensity had the greatest impact on biomass (Figure 3). The results of RSM (response surface methodology) showed that the optimal cultivation method for *E. pisciformis* AEW501 growth was determined to be a light intensity of 63 μmol photons m^−2^∙s^−1^, and a sodium acetate and diammonium phosphate concentration of 0.98 g L^−1^ and 0.79 g L^−1^, respectively. Under these conditions, the optimal theoretical value of biomass was 0.72 g L^−1^. To verify the reliability of the RSM experiment, three repeated experiments were tested on a photobioreactor (PBR) batch culture under optimal conditions and the biomass was 0.63 ± 0.06 g L^−1^, which was close to the theoretical value, indicating that the model was highly suitable.

A natural settlement was selected for the harvest process to minimize the cost. To determine the efficiency of the natural settlement, observation and measurement were performed. This study found that *E. pisciformis* cells could spontaneously sink to the bottom of the PBR system, and the settling efficiency reached 93.89% after aeration stopped for 80 min (Figure S3, Supporting Information), which suggested that *E. pisciformis* AEW501 would be harvested economically and efficiently in large-scale production.

### 2.2. Nutritional Composition

The nutritional composition of the proximate composition and nutritional profile of *E. pisciformis* AEW501 powder harvested from the PBR system was analyzed. The results showed that the protein content was 43.08 ± 1.73 g per 100 g dry cell weight, which was higher than polysaccharides and lipid. Additionally, the algal powder’s carbohydrate and crude lipid content were relatively equivalent, which were 21.46 ± 1.21 g and 25.84 ± 0.95 g per 100 g of algal powder, respectively. *E. pisciformis* AEW501 contained almost all the essential amino acids except cysteine and glutamine (Table 1). The content of glutamate (57.90 ± 8.63 g kg^−1^), aspartic acid (45.26 ± 3.44 g kg^−1^), leucine (44.88 ± 2.72 g kg^−1^), lysine (39.14 ± 3.97 g kg^−1^), and alanine (39.00 ± 2.41 g kg^−1^) was higher than other amino acids. The content of other amino acids such as threonine, serine, phenylalanine, and proline was nearly equal.

The fatty acid composition of *E. pisciformis* AEW501 was shown in Table 1; unsaturated fatty acids occupied 72.84% of total fatty acids. Linolenic acid, linoleic acid, eicosapentaenoic acid (EPA), and oleic acid, followed by docosahexaenoic acid (DHA) and arachidonic acid (ARA), and lesser heptadecenoic acid were contained in the *E. pisciformis* AEW501 cell. The essential trace elements such as iron, manganese, zinc, and copper were detected in the *E. pisciformis* AEW501 cell, and high phosphorus content (14,200 ± 282.84 mg kg^−1^) and potassium (6590 ± 353.55 mg kg^−1^) were also contained. Furthermore, algal cells contained vitamin A (β-carotene), vitamin E, and vitamin K in the concentrations of 197.00 ± 0.00 mg kg^−1^, 403.00 ± 0.00 mg kg^−1^, and 297.51 ± 4.90 μg kg^−1^, respectively. Vitamin B_1_ and vitamin D were observed at below detectable levels.

### 2.3. Paramylon Induced Culture

**Figure 4:**
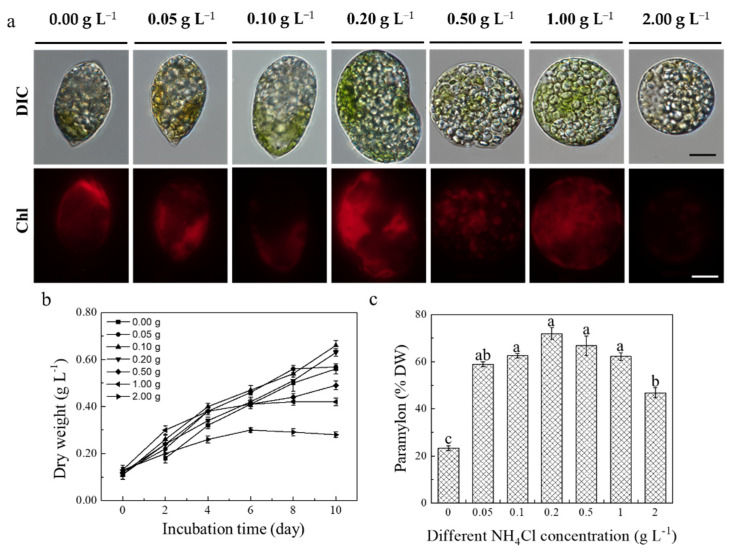
The induced effect of NH_4_Cl on the accumulation of paramylon in *E. pisciformis* AEW501 under mixotrophic culture conditions. (**a**) Intracellular paramylon changes in the algal cell under different concentrations of NH_4_Cl (scale bar: 10 μm). (**b**) Effects of different concentrations of NH_4_Cl on the growth of the algal cell. (**c**) The paramylon production of *E. pisciformis* AEW501 under different NH_4_Cl concentrations. Data are shown as mean ± standard deviation (*n* = 3), and different lowercase letters indicate a significant difference (*p* < 0.05).

The paramylon induction test indicated that NH_4_Cl had a significant promotion effect on *E. pisciformis* AEW501 paramylon content (Figure 4). The accumulation of paramylon granules in the *E. pisciformis* cytoplasm increased with the concentration of NH_4_Cl (Figure 4a). A significant concentration-dependent effect was found and the dry weight in the 0.10 and 0.20 g L^−1^ NH_4_Cl treatments was higher than in other treatment groups at the end of cultivation (*p* < 0.05). Similar to the dry weight, the maximum paramylon content (71.95%) was observed in the 0.20 g L^−1^ NH_4_Cl treatment (Figure 4c). However, cell viability was significantly reduced in the 1.00 and 2.00 g L^−1^ NH_4_Cl treatment groups with the shape becoming spherical cysts although the paramylon granules were visible. These results suggested that NH_4_Cl had a significant promoting impact on the accumulation of *E. pisciformis* polysaccharides. All further studies were conducted with the 0.2 g L^−1^ NH_4_Cl treatment based on these results.

### 2.4. Characterization of Paramylon

**Figure 5:**
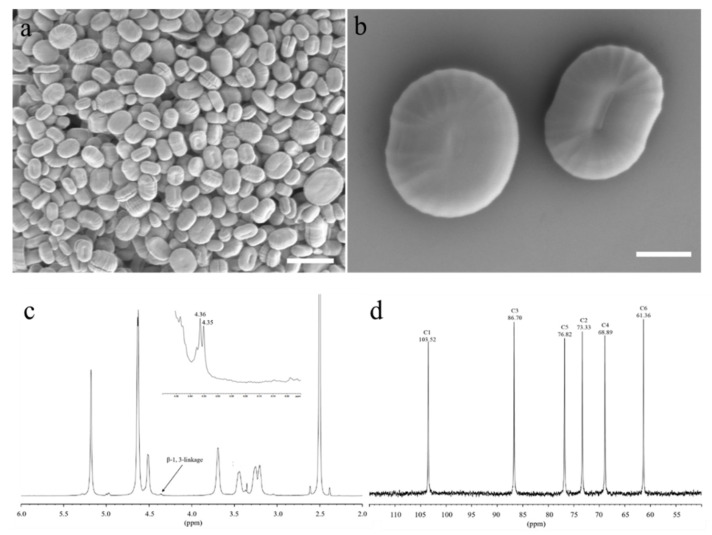
The characterization of paramylon. (**a**) The SEM of paramylon granules (scale bar: 5 μm). (**b**) The SEM of a granule showing the highly ordered structure of centrally radiated granules (scale bar: 1 μm). (**c**) ^1^H-NMR spectrum and (**d**) ^13^C-NMR spectrum of paramylon.

The crude paramylon of *E. pisciformis* AEW501 was extracted and the purity was determined to be 99.13 ± 0.61%. The SEM image (Figure 5a,b) confirmed that the extracted *E. pisciformis* AEW501 paramylon granules were very pure and free of impurities. Furthermore, the *E. pisciformis* AEW501 paramylon granules were (3.05 ± 2.14 μm) large discoidal granules with an erythrocytic-like surface appearance. The thickness was approximately 0.5–1.0 μm.

**Figure 6:**
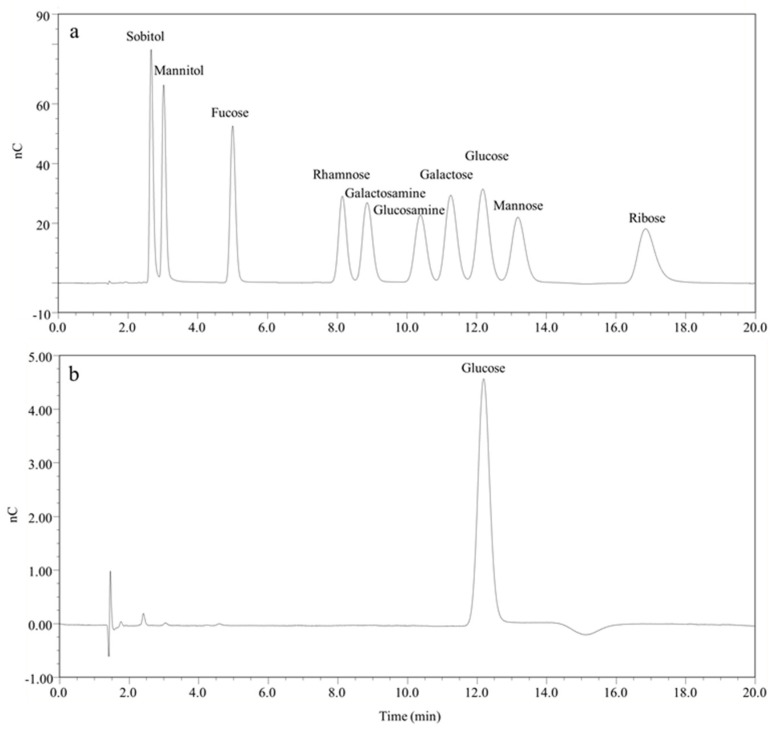
The monosaccharide composition analyses of purified paramylon from *E. pisciformis* AEW501. (**a**) The ion chromatography of the monosaccharide standard. (**b**) Acidolysis of paramylon.

To further determine the structure of *E. pisciformis* storage polysaccharides, ^1^H-NMR and ^13^C-NMR analyses were carried out. The ^1^H signal at 4.38 and 4.08 ppm was assigned the β-1,3-linkage and the β-1,6-linkage, respectively. The structural analysis indicated that *E. pisciformis* paramylon had pure β-1,3-linkage (Figure 5c). The ^13^C spectrum showed significant signals at 103.52, 86.70, 76.82, 73.33, 68.89, and 61.36 (Figure 5d), and in the range of 95–110 ppm, the paramylon had only one signal for the anomer carbon. After acidolysis treatments, ion chromatogram analysis indicated that glucose was the major monosaccharide of the *E. pisciformis* paramylon (Figure 6). All these results indicated that the *E. pisciformis* paramylon contained only one monosaccharide, i.e., β-1,3-glucan.

## 3. Discussion

Currently, there are three predominant methods for the large-scale cultivation of *Euglena*, as follows: (1) open systems (pond or raceway pond), (2) closed PBRs, and (3) fermenters. An open pond has been used for the commercial autotrophic cultivation of *E. gracilis* in Japan [30] and the open pond was proved as a low-tech method and cheaper when compared to PBRs and fermenters [31]. On the other hand, the dry weight of *E. gracilis* in the closed PBRs (5 L batch culture bioreactor) reached 23 g L^−1^ under heterotrophic cultivation with the supplementation of glucose and potato liquor [32]. The biomass of *E. gracilis* reached the peak (86.3 g L^−1^) under heterotrophic condition in a pilot-scale fermentation [21]. These cultural techniques could well support the large-scale cultivation of *Euglena.* However, the PBRs have not yet been involved in mixotrophic cultivation. This study established the optimal mixotrophic conditions of *E. pisciformis* AEW501 and tested it on a pilot-scale PBR.

The cultivation conditions optimization under mixotrophy is an available approach for achieving a high biomass of microalga. A recent study reported that a deep-sea diatom (*Chaetoceros*) could reached the maximum cell density at the optimal mixotrophic culture temperature and light intensity (15 °C, 20 μmol photons m^−2^∙s^−1^) [33]. Furthermore, Jin, Zhang, Zhou, Li, Hou, Xu, Chuai, Zhang, Han, and Hu [29] achieved an ultrahigh biomass (283.5 g L^−1^) of a unicellular green microalga *Scenedesmus acuminatus* using glucose as an organic source. *E. gracilis* could utilize glucose to reach the high biomass [21] and *E. gracilis* 815 achieved a high biomass (almost 0.9 g L^−1^) when ethanol was utilized under the mixotrophic mode [34]. In this study, *E. pisciformis* AEW501 was proved to metabolize sodium acetate as an organic carbon source to support mixotrophic growth; the optimal cultivation conditions were 20 °C, pH 7.0, and 63 μmol photons m^−2^∙s^−1^, and the concentrations of sodium acetate and diammonium hydrogen phosphate were 0.98 g L^−1^ and 0.79 g L^−1^, respectively. The maximal biomass was 0.72 g L^−1^ on the PBR culture system. This research suggested that high biomass could be obtained by optimizing the mixotrophic cultivation conditions.

Previous studies have focused on algae and bacteria co-cultivation strategies to improve paramylon production. In the *Vibrio natriegens* co-culture system, *E. gracilis* strain Z (Klebs CCALA 349) could reach the maximum paramylon content of 62.35% [18] and *E. gracilis* CCAP 1224/5Z paramylon content was up to 53.52% [19], which is lower than the data obtained in the present study. However, the paramylon yield of *E. gracilis* was approximately as high as 78.2% under the heterotrophic conditions in fermentation [21]. Moreover, the highest yield of paramylon (90% dry weight) was obtained from the WZSL white mutant *of E. gracilis* under controlled laboratory conditions [35], which is higher than the yield in this study.

Studies have shown that although salt stress might inhibit the growth of *E. gracilis*, it could significantly promote the accumulation of paramylon [17]. In the present study, NH_4_Cl was the only nitrogen source for the accumulation of paramylon in *E. pisciformis* cells under the mixotrophic mode. *E. pisciformis* cells with the supplementation of NH_4_Cl could accumulate plenty of paramylon granules. It could be deduced that salt stress conditions can induce paramylon accumulation in the *Euglena* cell.

The mechanisms leading to the increase in *E. pisciformis* AEW501 paramylon content under mixotrophic cultivation were speculated. Studies have shown that although salt stress might inhibit the growth of *E. gracilis*, it could significantly promote the accumulation of paramylon [17]. In the present study, although *E. pisciformis* cells with the supplementation of NH_4_Cl could accumulate plenty of paramylon granules, cell growth was significantly inhibited compared to the control group. Therefore, we speculated that the *Euglena* cell might induce paramylon accumulation under stress conditions. Furthermore, the β-1,3-glucan content was enhanced in *Poterioochromonas malhamensis* cells when NH_4_Cl was used as a nitrogen source [27]. On the other hand, a previous study reported that NH_4_Cl and sodium acetate as nitrogen and organic carbon sources could significantly increase *Scenedesmus obliquus* SXND-02 growth performance under mixotrophic conditions [36]. Notably, sodium acetate greatly enhanced *Euglena* cell growth depending on the glyoxylate pathway, alleviating the inhibition of a high concentration of NH_4_Cl [37]. Although these studies were suitable to explain our findings, further study would be needed to reveal the mechanisms of paramylon accumulation in *E. pisciformis* AEW501.

It is worth mentioning that nutrient composition data in this study indicated *E. pisciformis* has great potential industrial development value. *E. pisciformis* contains a life-like quantity of dietary protein, unsaturated fatty acids (EPA and DHA), nutritional vitamins, and minerals, which provides dietary nutritional support to human health. These data thus indicated that *E. pisciformis* AEW501 would be commercially valuable in the food and pharmaceutical industries. The characterization analysis revealed that the paramylon was composed of β-1,3-glucans and was extremely pure relative to other macromolecules (such as proteins and nucleic acids). On the other hand, this species was very easy to be harvested. Algal cells would be settled down to the bottom when the airlift facility stopped working, which could greatly save money on harvesting costs. All these results indicated *E. pisciformis* was worthy of exploitation.

## 4. Materials and Methods

### 4.1. Algal Strain and Culture Conditions

*E. pisciformis* AEW501 was obtained from the State Key Laboratory of Freshwater Ecology and Biotechnology in the Institute of Hydrobiology, Chinese Academy of Sciences (Wuhan, China). The inoculum was cultivated in a modified mixotrophic AF-6 medium (Table S1, Supporting Information) at 20 °C, with the constant light intensity of 50 μmol photons m^−2^∙s^−1^ using cool white fluorescent lamps as previously reported [25]. To prepare the inoculants, a single colony of *E. pisciformis* AEW501 was inoculated into a 50 mL modified AF-6 medium in a 100 mL Erlenmeyer flask.

### 4.2. Optimization of Cultural Conditions

After the pre-experiment screening, treatment groups of temperature (15 °C, 20 °C, and 25 °C), light intensity (15 µmol photons m^−2^∙s^−1^, 50 µmol photons m^−2^∙s^−1^, and 100 µmol photons m^−2^∙s^−1^), pH (6.05, 6.85, and 8.05), and photoperiod (10L:14D, 12L:12D, and 14L:10D) were set for the optimization experiment. In the nutrition optimization tests, sodium nitrate, urea, ammonium sulfate, tryptone, yeast extract, ammonium acetate, ammonium chloride, and diammonium phosphate as nitrogen sources were tested, and each was individually added to the modified AF-6 medium at 5 mM. Correspondingly, 10 mM of fructose, sodium acetate, ethanol, galactose, acetate, glycerin, glucose, sucrose, and α-Ketoglutaric acid as organic carbon sources were added to the modified AF-6 medium, respectively. The interaction between the factors was calculated by RSM using Design-Expert 8.0.6. The cell density was determined periodically using a hemocytometer (Thorma, Hirschmann, Germany). The relative growth was calculated according to the protocol of Morales-Sánchez et al. [38].

### 4.3. Airlift Photobioreactor (PBR) Cultivation

Algal cells in the exponential phase were inoculated into a 2 L flask with 600 mL of fresh modified AF-6 culture medium with sodium acetate as the organic carbon source and illuminated with 50 μmol photons m^−2^∙s^−1^ light intensity for ten days, then transferred to a 5 L glass jar with 3 L of medium at an aeration rate of 1 L min^−1^. The entire algal suspension was inoculated into a 120 L airlift PBR system (Photon System Instruments, Czech). The cultivation conditions were as follows: 20 °C temperature, 100 μmol photons m^−2^∙s^−1^ light intensity, which was provided by LED lamps, constant pH (7.00), and 3 L min^−1^ aeration. The culture temperature was automatically controlled by a heating rod or refrigeration equipment, and pH was maintained automatically by adding 1 M HCl or 1 M NaOH solution. During the PBR operation, dissolved oxygen (DO) was held between 8.0–9.0 mg L^−1^, and the water level sensor controlled the total volume of the culture system. When algal cells reached the stationary phase, the cultures were harvested by natural sedimentation and centrifugation. The pellet was washed with deionized water before being freeze-dried with a vacuum freeze dryer (CTFD-12S, China). Finally, the freeze-dried algal powder was stored at −20 °C for nutritional component analysis.

### 4.4. Nutritional Component Determination

The protein content of the algal powder was determined using the modified Bradford Method [39] with the Bradford protein quantification kit (Nanjing Vazyme Biotech Co., Ltd., Nanjing, China). First, 10 mg of freeze-dried algal powder was weighed and 0.1 mL of 1 mol L^−1^ NaOH was added before incubating at 80 °C for 10 min. Then, 0.9 mL of sterilized water was added before centrifuging at 10,000 rpm 4 °C for 10 min and then the supernatant was kept. These steps were repeated two or more times before combining all the supernatants into one tube. The ratio of the extract to working solution was 1:10, and then the samples were incubated at room temperature for 10 min. The protein content was quantified using the Multimode Plate Reader (PerkinElmer, VICTOR Nivo, America) at 595 nm with the standard curve.

The carbohydrate content was measured with minor modification by the phenol sulfuric acid method [40]. Briefly, we weighed 10 mg algal powder in a 2.0 mL centrifuge tube before adding 0.5 N H_2_SO_4_, and then a cryo-disintegrator to the broken algal cells before centrifugation (10,000 rpm for 5 min). Repeat these steps three times, and then combine all the supernatants into a 50 mL volumetric flask as a sample. The carbohydrate content was calculated according to the standard curve of glucose.

The total lipid content and fatty acid composition were determined according to the protocol of Fan et al. [25]. The range of vitamins in *E. pisformis* cells was determined according to Syad et al. [41]. The mineral content was performed using an atomic absorption spectrophotometer (Perkin Elmer Analyst 800 with the flame furnace) following the study of Sakthivel [42]. The amino acid profiles were estimated according to the Pico-Tag method [43]. The samples were hydrolyzed with HCl, and post-column derivatization with ninhydrin was then detected with an Amino Acid Analyzer (A300, MembraPure Bodenheim, Frankfurt, Germany).

### 4.5. Paramylon Induced Culture

The mixotrophic inoculum was harvested in the logarithmic phase by centrifugation at 3000 rpm for 5 min and incubated into 500 mL Erlenmeyer flasks with a 240 mL sterilized medium. For the paramylon induction test, different concentrations of NH_4_Cl were added to the modified nitrogen-free AF-6 medium. The medium was supplemented with 0.00, 0.05, 0.10, 0.50, 1.00, and 2.00 g L^−1^ NH_4_Cl during the inducing test, and stocks were sterilized by a 0.22 μm pinhole filter before being added into the medium, finally making the final working volume of 250 mL medium in 500 mL flasks. All flasks were shaken evenly by hand 4–5 times a day to promote gas exchange and avoid algal cells adhering to the bottom. Each treatment group was carried out in three independent biological repeats.

### 4.6. Paramylon Extraction and Quantification

Freeze-dried algal powder with 20 mg (M_1_) was weighed, washed with 4 mL acetone, and centrifugated at 4000 rpm for 5 min to remove the lipophilic compounds. The supernatant was discarded, subsequently 1% SDS was added to the sediment, then it was transferred to a 1.5 mL centrifuge tube before being heated at 85 °C for 30 min. Then sample was centrifuged at 12,000 rpm for 5 min, and the sediment was crude paramylon. The extracted paramylon was refined twice with 500 g L^−1^ urea solution and once with 1 mL dd H_2_O. Finally, the pure paramylon was dried at 50 °C and weighted (M_2_) before being dissolved in 0.5 mol L^−1^ NaOH solution for determination [44].

The paramylon content was tested according to Dubois et al. [45]. The paramylon granule was dissolved in 0.50 mol L^−1^ NaOH before dilution. An aliquot (0.5 mL of 6% phenol and 2.5 mL of concentrated sulfuric acid) was added to the sample successively, then the mixture was vortexed and reacted at room temperature for 20 min before measuring the absorbance at 490 nm with a UV-visible spectrophotometer (UV1780, Shimadzu, Kyoto, Japan). The paramylon (M_3_) content was calculated according to the standard curve of glucose. The production of paramylon (P, %) was calculated as P = M_2_/M_1_ × 100%, and the purity of paramylon was calculated from M_3_/M_2_.

### 4.7. Characterization of E. pisciformis Paramylon

#### 4.7.1. Scanning Electron Microscopy (SEM)

The pure paramylon was placed on a monocrystalline silicon wafer soaked with 0.01 mg mL^−1^ polylysine. It was subjected to natural air-drying before the silicon wafer was fixed on the sample stage with conductive glue and an ion sputter (Hitachi E-1010, Hitachi Ltd., Tokyo, Japan) was used to coat the sample with a conductive film. The sample was visualized by a scanning electron microscope (Hitachi S4800, Hitachi Ltd., Tokyo, Japan) and micrographs were recorded.

#### 4.7.2. Monosaccharide Composition and Structure Analysis

The purified sample was treated by acidolysis. The hydrolysates were analyzed using an ion chromatogram analysis system [46]. The dried paramylon granules (20 mg) were dissolved in DMSO-*d*_6_ for NMR measurements. ^1^H-NMR (600 MHz) and ^13^C-NMR (600 MHz) spectra were recorded at 25 °C (298 K) on a Bruker 600 MHz NMR spectrometer (Bruker Biospin, Rheinstetten, Germany). For ^1^H-NMR experiments, the parameters were as follows: a spectral width of 9.0 kHz, a 90 °C pulse, an acquisition time of 3.6 s, and a relaxation delay of 3 s for 8 scans. The corresponding ^13^C-NMR parameters were as follows: a spectral width of 12.1 kHz, a 30 °C pulse, an acquisition time of 0.7 s, and a relaxation delay of 3 s for 2048 scans. In all the cases, signals were referenced to the internal DMSO at 2.50 ppm for ^1^H-NMR and 39.51 ppm for ^13^C-NMR experiments, respectively.

### 4.8. Statistical Analysis

All the parameters were performed in three replicates. The data were analyzed using a one-way analysis of variance with the least-significant difference (LSD) multiple comparison test (SPSS 22.0, Chicago, IL, USA). The statistically significant difference was considered when *p* < 0.05.

## 5. Conclusions

This study established the optimal mixotrophic cultivation for *E. pisciformis* AEW501 biomass and paramylon production under the mixotrophic mode in a pilot-scale PBR. The paramylon yield reached the maximum (71.39%) under mixotrophic conditions with the 0.2 g L^−1^ NH_4_Cl treatment. Paramylon was identified as composed of pure β-1,3-glucans with a high purity of 99.13 ± 0.61%. The nutrients and beneficial bioactive substances component in the algal powder indicated that this species possesses huge potential for industrial exploitation.

## Figures and Tables

**Table 1:** Component analysis of *E. pisciformis* AEW501 freeze-dried powder.

Component	Unit	Content	Component	Unit	Content
Protein	g kg^−1^	430.82 ± 17.37	C18:2n6	% (fatty acid)	7.90 ± 0.94
Carbohydrate	g kg^−1^	214.60 ± 12.11	C18:3n3	% (fatty acid)	20.01 ± 3.37
Crude lipid	g kg^−1^	258.49 ± 9.52	C20:2	% (fatty acid)	2.06 ± 0.31
Aspartic acid	g kg^−1^	45.26 ± 3.44	C20:3n3	% (fatty acid)	1.46 ± 0.54
Threonine	g kg^−1^	25.32 ± 2.51	C20:4n6	% (fatty acid)	4.16 ± 0.97
Serine	g kg^−1^	22.17 ± 1.80	C20:5n3	% (fatty acid)	5.81 ± 1.09
Glutamate	g kg^−1^	57.90 ± 8.63	C22:6n3	% (fatty acid)	6.74 ± 1.34
Glycine	g kg^−1^	29.13 ± 3.34	Total unsaturated fatty acid	%	72.84 ± 5.39
Alanine	g kg^−1^	39.00 ± 2.41	β-carotene	mg kg^−1^	197.00 ± 0.00
Valine	g kg^−1^	30.54 ± 1.96	Vitamin B_1_	mg kg^−1^	ND (<0.10)
Methionine	g kg^−1^	8.00 ± 0.21	Vitamin B_2_	mg kg^−1^	0.51 ± 0.00
Isoleucine	g kg^−1^	20.45 ± 0.93	Vitamin D	μg kg^−1^	ND (<0.70)
Leucine	g kg^−1^	44.88 ± 2.72	Vitamin E	mg kg^−1^	403.00 ± 0.00
Tyrosine	g kg^−1^	17.37 ± 0.85	Vitamin K	μg kg^−1^	297.51 ± 4.90
Phenylalanine	g kg^−1^	26.00 ± 4.54	Calcium	g kg^−1^	3.14 ± 0.02
Lysine	g kg^−1^	39.14 ± 3.97	Iron	g kg^−1^	3.49 ± 0.06
Histidine	g kg^−1^	10.98 ± 1.46	Potassium	g kg^−1^	6.59 ± 0.35
Arginine	g kg^−1^	345.21 ± 39.17	Magnesium	g kg^−1^	3.99 ± 0.02
Proline	g kg^−1^	208.10 ± 26.44	Phosphorus	g kg^−1^	14.20 ± 0.28
C16:0	% (fatty acid)	5.54 ± 9.74	Selenium	mg kg^−1^	0.13 ± 0.00
C16:1	% (fatty acid)	11.30 ± 13.17	Zinc	mg kg^−1^	147.50 ± 0.71
C17:0	% (fatty acid)	1.25 ± 0.36	Sodium	mg kg^−1^	823.50 ± 4.95
C17:1	% (fatty acid)	0.77 ± 0.04	Copper	mg kg^−1^	6.03 ± 0.13
C18:1n9	% (fatty acid)	5.84 ± 0.39	Manganese	mg kg^−1^	138.50 ± 0.71

The results were represented as mean ± standard deviation (*n* = 3). ND represented not detected.

## Data Availability

The data presented in this study are available in this article.

## Supplementary Materials

The following supporting information can be downloaded at: https://www.mdpi.com/article/10.3390/md20080518/s1. Figure S1: The optimization of diammonium phosphates and sodium acetate concentration (a: Diammonium phosphates; b: Sodium acetate); Figure S2: Natural sedimentation of *E. pisciformis* AEW501 during harvest in a 120 L PBR (Stop ventilation time: A: 0 min; B: 5 min; C: 20 min; D: 40 min; E: 60 min); Figure S3: The settling efficiency of *E. pisciformis* AEW501 during harvest; Table S1: Composition of modified AF-6 medium.
