# Supplementary material for: Mixotrophic Cultivation Optimization of Microalga Euglena pisciformis AEW501 for Paramylon Production
Source: Mar Drugs. 2022 Aug 14;20(8):518. doi: 10.3390/md20080518 (PMC9410504; doi:10.3390/md20080518)
Supplement: Supplementary file 1 [file marinedrugs-20-00518-s001.zip › marinedrugs-1854136-supplementary.pdf]

Table S1. Composition of modified AF-6 medium

Components	Content (g/L)	Components	Content (g/L)	Components	Content (g/L)
CH_3COONa	1.00	$MgSO_4 \cdot 7H_2O$	0.3	Ferric citrate	0.002
$NaNO_3$	0.14	Thiamine HCl	0.1	Na_2EDTA	0.75
$(NH_4)_2HPO_4$	0.20	Vitamin B6	0.1	$MnCl_2 \cdot 4H_2O$	0.041
K_2HPO_4	0.005	Biotin	0.2	$ZnCl_2 \cdot 7H_2O$	0.005
KH_2PO_4	0.01	Vitamin B12	0.1	$Na_2MoO_4 \cdot 2H_2O$	0.004
$CaCO_3$	0.01	Citric acid	0.002	$FeCl_3 \cdot 6H_2O$	0.097
$CaCl_2 \cdot 2H_2O$	0.01	$CoCl_2 \cdot 6H_2O$	0.002	-	-

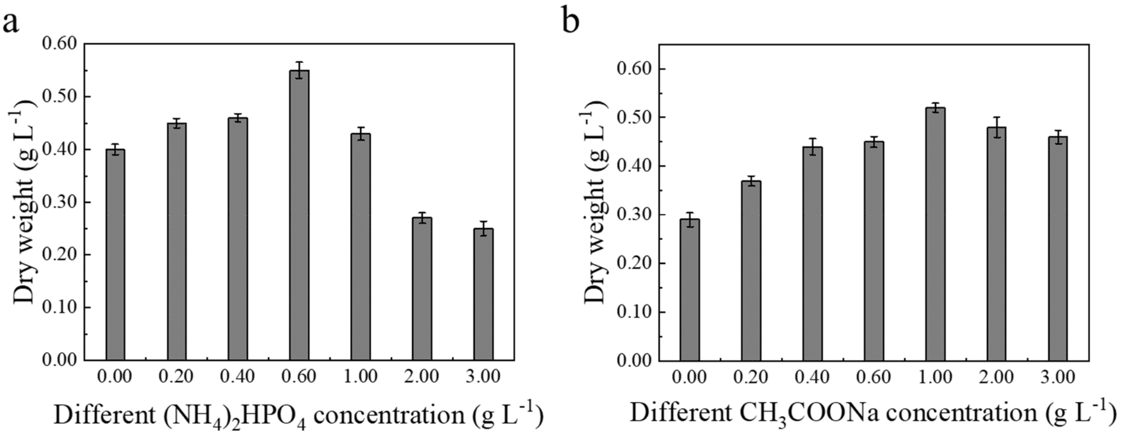

Figure S1. The optimization of diammonium phosphates and sodium acetate concentration after 14 days cultivation. (a: Diammonium phosphates; b: Sodium acetate.) Data were shown as mean ± standard deviation (n = 3).

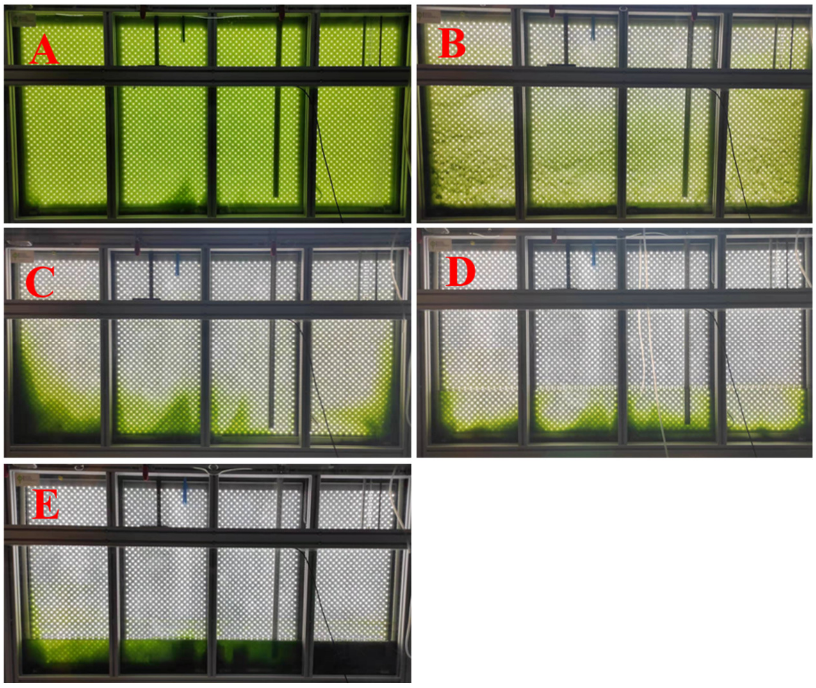

Figure S2. Natural sedimentation of *E. pisciformis* AEW501 during harvest in a 120 L PBR. (Stop ventilation time: A: 0 min; B: 5 min; C: 20 min; D: 40 min; E: 60 min.)

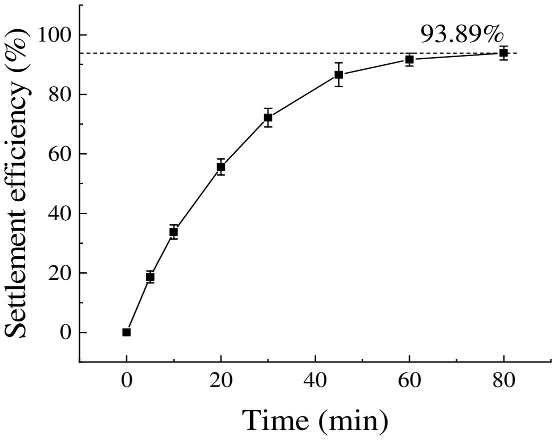

Figure S3. The settling efficiency of E. pisciformis AEW501 during harvest.
